# Beyond Da Vinci: Comparative Review of Next-Generation Robotic Platforms in Urologic Surgery

**DOI:** 10.3390/jcm14196775

**Published:** 2025-09-25

**Authors:** Stamatios Katsimperis, Lazaros Tzelves, Georgios Feretzakis, Themistoklis Bellos, Panagiotis Triantafyllou, Polyvios Arseniou, Andreas Skolarikos

**Affiliations:** 1 Second Department of Urology, National and Kapodistrian University of Athens, Sismanogleio Hospital, 15126 Athens, Greece; 2 School of Science and Technology, Hellenic Open University, 26335 Patras, Greece; 3 Department of Urology, Red Cross General Hospital of Athens, 11526 Athens, Greece

**Keywords:** robotic surgery, urologic oncology, novel robotic platforms, next-generation robotic platforms, Hugo RAS, Versius, Avatera, Toumai, Dexter, Hinotori

## Abstract

Robotic surgery has become a cornerstone of modern urologic practice, with the da Vinci system maintaining dominance for over two decades. In recent years, however, a new generation of robotic platforms has emerged, introducing greater competition and innovation into the field. These systems aim to address unmet needs through features such as modular architectures, enhanced ergonomics, haptic feedback, and cost-containment strategies. Several platforms—including Hugo™ RAS, Versius™, Avatera™, REVO-I, Hinotori™, Senhance™, KangDuo, MicroHand S, Dexter™, and Toumai^®^—have entered clinical use with early results demonstrating perioperative and short-term oncologic outcomes broadly comparable to those of established systems, particularly in procedures such as radical prostatectomy, partial nephrectomy, and radical cystectomy. At the same time, they introduce unique advantages in workflow flexibility, portability, and economic feasibility. Nevertheless, important challenges remain, including the need for rigorous comparative trials, standardized training curricula, and long-term cost-effectiveness analyses. The integration of artificial intelligence, augmented reality, and telesurgery holds the potential to further expand the role of robotics in urology, offering opportunities to enhance precision, improve accessibility, and redefine perioperative care models. This review summarizes the evolving landscape of robotic platforms in urology, highlights their clinical applications and limitations, and outlines future directions for research, training, and global implementation.

## 1. Introduction

Robotic-assisted surgery has revolutionized the field of minimally invasive urology, combining enhanced dexterity, tremor filtration, and three-dimensional visualization to allow precise maneuvers in confined anatomical spaces [1]. The journey toward modern robotic systems began in the late 1980s with pioneering prototypes such as the PUMA 560, initially applied for stereotactic neurosurgical biopsies, and the PROBOT, developed for transurethral resection of the prostate. These early devices were highly specialized and lacked the flexibility to perform a broad range of surgical procedures [2]. In the 1990s, systems like ROBODOC, designed for orthopedic applications, demonstrated the potential for computer-assisted precision but were far from the multi-specialty platforms we know today [3]. The real breakthrough came with the da Vinci Surgical System, introduced by Intuitive Surgical and cleared by the U.S. Food and Drug Administration in 2000 [2]. Combining wristed instruments with seven degrees of freedom, immersive 3D high-definition vision, and motion scaling, da Vinci allowed surgeons to perform complex urologic procedures with unparalleled precision. Radical prostatectomy, in particular, became a defining procedure for robotic surgery, rapidly replacing open approaches in many high-volume centers [4,5]. Over the following two decades, da Vinci became synonymous with robotic surgery, supported by a vast evidence base demonstrating its safety, efficacy, and favorable functional outcomes [6,7]. However, the monopoly created by patent protections and high market entry barriers also led to significant limitations. The high acquisition cost—often exceeding two million US Dollars—along with expensive maintenance contracts and proprietary instruments, restricted access to well-funded institutions [8]. Smaller hospitals and resource-limited settings were left without realistic options for integrating robotic surgery into their programs. The expiration of key patents, coupled with growing global demand for minimally invasive surgery, created the conditions for a new wave of competition. Today, several manufacturers worldwide have introduced multiport robotic platforms designed to challenge the dominance of da Vinci. These systems vary in their engineering philosophy, ergonomics, instrument design, and economic models. Some emphasize modularity and portability, others focus on haptic feedback or hybrid laparoscopic–robotic workflows, while certain manufacturers aim to disrupt costs through reusable instruments or single-use devices. This diversification represents a pivotal moment in the evolution of robotic surgery. Increased competition has the potential to drive technological innovation, reduce costs, and broaden access. At the same time, it presents new challenges for healthcare systems, surgeons, and policymakers, who must evaluate these platforms based on safety, performance, cost-effectiveness, and compatibility with existing infrastructure.

This narrative review aims to provide a comparative overview of the most prominent emerging robotic platforms in urology, focusing on their design features, clinical applications, reported outcomes, and practical considerations for adoption. By critically examining each system alongside the established da Vinci benchmark, we aim to guide decision-making in this rapidly evolving technological landscape.

## 2. Methods

### Search Strategy

A comprehensive literature search was conducted to identify publications on next-generation robotic platforms in urologic surgery. Searches were performed in PubMed/MEDLINE, Scopus, Web of Science and Google Scholar from database inception to August 2025. The following keywords and their combinations were used: “robotic surgery,” “next-generation robotic platforms,” “urology,” “Hugo RAS,” “Versius,” “Senhance,” “Avatera,” “REVO-I,” “MicroHand,” “Dexter,” “Toumai,” “Hinotori,” and “surgical robotics.” Relevant articles were selected based on their focus on technical design, clinical applications, early outcomes, or economic considerations of these systems. Preference was given to peer-reviewed original studies, case series, and reviews published in English. Reference lists of key articles were also manually reviewed to identify additional relevant publications. The decision to perform a narrative rather than systematic review was based on the heterogeneity of the literature, the rapid pace of technological development, and the presence of predominantly early-phase data for many systems. While this approach allows for a broader contextual discussion, it also carries the limitation of potential selection bias. Where possible, information was synthesized from peer-reviewed clinical data, manufacturer specifications, and expert commentary to provide a balanced assessment of each platform. The key design characteristics and unique features of the most prominent next-generation robotic platforms identified through this search are summarized in Table 1.

## 3. Overview of Next-Generation Robotic Platforms

### 3.1. Hugo™ RAS (Medtronic)

#### 3.1.1. Design and Technical Features

The Hugo™ Robotic-Assisted Surgery system, developed by Medtronic (Galway, Ireland), represents one of the most significant recent entrants into the robotic surgery market. Designed with an open-console architecture and modular, wheeled robotic arms, Hugo™ emphasizes flexibility, portability, and adaptability to different operating room layouts. Each arm functions as an independent unit, mounted on a mobile cart that can be positioned according to the surgical approach, patient anatomy, and available space. This configuration contrasts with the fixed patient-side cart of da Vinci xi (Intuitive Surgical, Sunnyvale, CA, USA), potentially enhancing versatility, especially in multi-specialty hospitals. The open console provides high-definition three-dimensional visualization through a head-up display while allowing direct eye contact and verbal communication with the surgical team [9]. Surgeons operate using two arm-controllers with a pistol-like griphand designed for intuitive instrument manipulation, with wristed instruments offering seven degrees of freedom and motion scaling for fine dissection [10]. The system accommodates advanced energy devices and supports multi-quadrant surgery without the need for patient repositioning in many cases. In urology, Hugo™ RAS has been applied to radical prostatectomy, partial nephrectomy, radical cystectomy and other non-oncology procedures.

#### 3.1.2. Clinical Applications and Outcomes

Prostatectomy has generated the strongest body of evidence for the Hugo™ platform, with outcomes that consistently parallel those of da Vinci. A pivotal comparative series from Bravi and colleagues evaluated 542 men undergoing robot-assisted radical prostatectomy (RARP) at a high-volume European center, including 164 procedures performed with Hugo [11]. Despite a higher proportion of palpable disease in the Hugo cohort (34% vs. 25%), perioperative outcomes were indistinguishable from da Vinci: operative time differed by only 16 min (*p* = 0.12), blood loss was virtually identical (+3 mL; *p* = 0.9), and complication rates remained comparable (Clavien–Dindo ≥2: OR 1.66; *p* = 0.5) [11]. Oncologic control was preserved, with positive surgical margins reported in 12% of Hugo cases versus 15% of da Vinci cases, and early continence recovery was equivalent at both one month (OR 0.78; *p* = 0.4) and three months (OR 1.17; *p* = 0.7). Andrade’s multicenter Brazilian experience with 100 consecutive cases reinforced the platform’s reproducibility, reporting a median operative time of 185 min, blood loss of 200 mL, and continence recovery in 85% of men by three months, with a PSM rate of 22% [12]. In Japan, Takahara and colleagues described their first 30 Hugo RARPs with mean operative time of 240 min, blood loss of 150 mL, PSMs in 13%, and continence recovery in 83% of patients by three months [13]. Italian data from Totaro et al. further support these findings, with 50 Hugo cases yielding a median operative time of 200 min, blood loss of 250 mL, PSMs in 14%, and 86% continence recovery at three months, without conversions or major complications [14]. Complementing these single-center series, Antonelli’s multicenter European study confirmed comparable perioperative outcomes and functional recovery across different institutions, further validating the generalizability of Hugo for prostatectomy [15]. Taken together, these reports demonstrate that the platform delivers perioperative safety, oncological control, and functional results that match da Vinci, even in early adoption phases across diverse healthcare systems.

Renal surgery with the Hugo™ system has been increasingly investigated, particularly in the field of partial nephrectomy where precision and ischemia control are critical. Bobrowski and colleagues presented one of the largest early series with 49 patients undergoing robot-assisted partial nephrectomy (RAPN), reporting a median operative time of 150 min, warm ischemia time of 16 min, and blood loss of 200 mL [16]. The trifecta outcome—defined as negative margins, warm ischemia <25 min, and absence of major complications—was achieved in 71% of cases, with only one Clavien–Dindo ≥3 event, underscoring the platform’s safety profile [16]. Chierigo and colleagues detailed their first 10 RAPNs with Hugo, including two retroperitoneal and eight transperitoneal procedures, highlighting trocar placement, docking strategies, and stepwise technical execution; all procedures were completed successfully, confirming feasibility in both surgical approaches during early adoption [17]. Gaya and colleagues provided further technical validation of the retroperitoneal approach, describing three RAPNs with warm ischemia between 16 and 22 min and blood loss of 50–250 mL, all completed without conversion or intraoperative complications [18]. Prata et al. offered one of the first comparative studies, analyzing 27 Hugo RAPNs versus 62 laparoscopic partial nephrectomies performed by the same experienced surgeon [19]. Patients in the Hugo group experienced significantly shorter operative times (92 vs. 149.5 min, *p* = 0.005) and reduced hospital stay (3 vs. 5 days, *p* = 0.002), with similar rates of positive surgical margins (3.7% vs. 4.8%). Trifecta achievement favored Hugo RAPN (92.6% vs. 82.3%), although the difference did not reach statistical significance [19]. Expanding beyond nephrectomy, Morizane et al. reported the world’s first experience of robot-assisted radical nephroureterectomy (RANU) using Hugo, in five patients treated for upper tract urothelial carcinoma [20]. After optimizing docking configurations, all procedures were completed transperitoneally, with a median operative time of 283 min (robotic console time 187 min), minimal blood loss (median 20 mL), no transfusions, and no Clavien–Dindo ≥3 complications [20]. Outcomes were comparable to those achieved with da Vinci Xi, confirming the platform’s technical feasibility in complex upper tract surgery.

Experience with Hugo™ RAS in radical cystectomy is limited but encouraging. Rocco et al. reported the first two cases, including one intracorporeal neobladder and one ureterostomy, both completed without intraoperative complications; docking required ~35 min and console time ~140–150 min [21]. Gaya et al. added two further cases with ileal conduit diversion, showing operative times of 360–420 min, blood loss of 200–400 mL, and no positive margins. Only one patient developed a minor ileus [22].

Non-oncologic and reconstructive urology has also benefited from the introduction of Hugo with Rebuffo et al. reporting the first series of 10 pyeloplasties performed at a tertiary referral center [23]. Median docking time was only 8 min, while console time averaged 89.5 min. No conversions or major complications occurred, and only one minor technical issue was noted, which did not compromise surgical success [11]. These early results suggest that Hugo™ can be safely and effectively used for minimally invasive pyeloplasty, providing favorable perioperative outcomes comparable to established robotic platforms.

Comparative studies with da Vinci provide the most robust evidence of Hugo’s clinical equivalence. Head-to-head analyses, have demonstrated no significant differences in oncological or functional outcomes, including margin status, biochemical recurrence, continence, and potency recovery [24,25,26,27]. Some studies noted slightly longer vesicourethral anastomosis or console times during radical prostatectomy with Hugo™, particularly among surgeons early in their adoption curve [26]. However, overall perioperative safety, complication profiles, and patient-centered outcomes were preserved. Importantly, surgeons with prior da Vinci experience transitioned more efficiently to Hugo™, while those new to robotic platforms still achieved acceptable outcomes, suggesting that the learning curve is manageable [27]. These findings underscore that differences in efficiency reflect surgeon adaptation rather than limitations of the system itself.

### 3.2. Versius™ (CMR Surgical)

#### 3.2.1. Design and Technical Features

The Versius™ Surgical Robotic System, developed by UK-based CMR Surgical (Cambridge, UK), is built around a philosophy of modularity and ergonomic optimization. Each of its three or four robotic arms is mounted on a compact, wheeled bedside unit, enabling flexible positioning around the patient and adaptability to different port configurations [28]. This modular approach allows the system to be deployed in a variety of operating room environments without requiring extensive structural modifications. For hospitals without dedicated robotic theaters, the ability to store the arms between cases is a distinct logistical advantage. The console is open, with the surgeon seated in an ergonomic posture that allows communication with the rest of the surgical team. The hand controllers are modeled to feel similar to traditional laparoscopic instruments, making the transition from laparoscopy potentially smoother and reducing the cognitive load for surgeons accustomed to conventional minimally invasive techniques [29]. The system delivers three-dimensional high-definition visualization and uses fully wristed instruments with seven degrees of freedom for complex dissection and suturing [28].

#### 3.2.2. Clinical Applications and Outcomes

The clinical application of the Versius™ robotic system in urology was preceded by structured preclinical evaluation. In cadaveric and porcine models, Thomas et al. demonstrated the feasibility of completing radical nephrectomy, radical prostatectomy (including Retzius-sparing), and pelvic lymph node dissection with successful procedural completion, adequate surgical access, and no device-related complications [30]. These findings established the foundation for subsequent clinical translation. The first human case of RARP with Versius, reported by Rocco et al., confirmed technical feasibility, safe perioperative recovery, and negative surgical margins [31]. This early experience was expanded by De Maria et al., who reported on 18 consecutive RARPs, achieving a median operative time of 213 min, no conversions, low perioperative morbidity, and continence recovery in 72% of patients at two months [32]. Polom et al. further evaluated 58 RARPs in a center without prior robotic experience, observing a conversion rate of 0.6%, median blood loss of 437 mL, a positive margin rate of 25.8%, and continence recovery exceeding 90% at 12 months [33]. Broader applicability was demonstrated in the largest series to date by Hussein et al., encompassing 106 procedures across benign and malignant indications, including nephrectomy, pyeloplasty, stone surgery, and cystectomy [34]. This cohort reported median blood loss of 123 mL, a 6% conversion rate (mainly due to adhesions), and an 8% incidence of high-grade complications, with perioperative outcomes largely comparable to matched procedures performed with the da Vinci system, aside from a modest reduction in operative time for partial nephrectomy using Versius [34]. Taken together, these preclinical and clinical experiences suggest that the Versius platform delivers perioperative, oncologic, and functional outcomes broadly consistent with established robotic systems, while its modular design and open-console ergonomics introduce distinctive workflow considerations. Longer-term oncologic follow-up and multi-institutional studies remain necessary to define its definitive role in contemporary urological practice.

### 3.3. Avatera™ (Avateramedical GmbH)

The Avatera™ Surgical System, developed in Germany by Avateramedical GmbH (Jena, Germany), distinguishes itself with its exclusive use of single-use instruments. This design eliminates the need for reprocessing and sterilization between cases, potentially reducing turnover times and the risk of cross-contamination. While this approach shifts cost considerations toward consumables, it may appeal to hospitals seeking to simplify workflow and reduce infrastructure requirements for instrument reprocessing and might also eliminate potential damage costs [35]. The system employs a closed-console design with immersive three-dimensional high-definition vision, allowing surgeons to work in a fully focused, distraction-free environment. The console ergonomics are similar to those of the da Vinci system, which may reduce the learning curve for surgeons already experienced in robotic surgery. The patient-side cart supports four robotic arms, each with fully wristed instruments capable of fine articulation and precise tissue handling.

The introduction of the Avatera™ Surgical System into urology has been preceded by a series of preclinical and translational investigations with current evidence originating almost exclusively from the Professor Liatsikos group. Martinez-Ballesta et al. demonstrated feasibility in porcine models, successfully performing nephrectomy, pyeloplasty, and cystectomy without major intraoperative complications, thereby validating the platform’s basic technical capabilities [36]. Subsequent experimental work by Anaplioti et al. explored the learning curve in a dry-lab setting, showing that trainees achieved proficiency in fundamental skills (peg transfer, cutting, suturing) more rapidly with Avatera compared to laparoscopy, particularly when prior laparoscopic experience was present [37]. Haney et al. expanded this assessment within the IDEAL-D Phase 0 framework, completing 23 upper and lower urinary tract procedures in cadaveric and porcine models with high surgeon satisfaction scores and favorable performance metrics on validated assessment tools, further confirming reproducibility and safety in preclinical conditions [38]. Clinical translation was first reported by Kallidonis et al., who presented a prospective series of nine robot-assisted pyeloplasties for ureteropelvic junction obstruction [39]. All procedures were successfully completed without intraoperative or postoperative complications, with a median console time of 88 min, minimal blood loss, and no conversions. Postoperative recovery was uneventful in all cases, and functional follow-up confirmed unobstructed drainage with resolution of symptoms. More recently, Gkeka et al. provided the most comprehensive early clinical experience, describing 14 extraperitoneal RARPs with successful completion in all cases, median draping 9.5 min (7–13), docking 10 min (5–40), console 103.5 min (90–121), no conversions or transfusions, median hemoglobin drop 1.95 g/dL, positive margins in 2 patients, and at 6 months 78.6% continence with erections adequate for intercourse in 77.7% of nerve-sparing patients [40]. Collectively, these data—exclusively generated by the Liatsikos group—indicate that Avatera is a feasible and safe robotic platform for both oncologic and reconstructive urologic surgery, with early perioperative outcomes comparable to established systems. Nevertheless, its definitive positioning will require larger, multi-institutional studies with longer follow-up and comparative cost-effectiveness analyses.

### 3.4. REVO-I (Meere Company Inc.)

REVO-I, produced by South Korea’s Meere Company Inc. (Hwaseong/Gyeonggi-do, South Korea)., represents the first domestically developed multiport robotic platform in the country. Designed to closely resemble earlier da Vinci models in terms of console layout and instrument control, REVO-I aims to reduce the learning curve for surgeons already trained on established systems. The closed-console design provides immersive 3D HD visualization, while its multi-jointed instruments allow fine dissection in confined spaces. The patient-side cart supports four robotic arms, with flexible port placement options [9]. REVO-I’s instruments are reusable, offering potential cost savings in high-volume centers. Maintenance and acquisition costs are generally lower than those of international competitors, making it attractive to resource-conscious healthcare systems.

The clinical development of the REVO-I system was preceded by preclinical work. In a porcine feasibility study, Kim et al. successfully performed four robot-assisted partial nephrectomies with REVO-I, reporting mean docking and operative times of 12.3 and 36.3 min, respectively, mean warm ischemia time of 13 min, and no intraoperative complications or conversions, thus validating its technical safety and basic performance [41]. Building on this, Chang et al. described the first clinical use in humans, conducting three robot-assisted radical prostatectomies [42]. All procedures were completed without conversion, with operative times ranging from 163 to 200 min and minimal blood loss (200–300 mL), and all patients were discharged without major complications [42]. Longer-term functional outcomes were not reported, but perioperative feasibility was confirmed. More robust evidence was provided by Alip et al. in a propensity-matched comparison of 33 patients treated with REVO-I versus 33 with the da Vinci Si system [43]. Perioperative outcomes were broadly similar, with no conversions in either group, comparable complication rates (~9%), and equivalent margin positivity (48% vs. 45%). Functional recovery was also similar, with continence at three months achieved in nearly three-quarters of patients in both arms. The main difference was procedural efficiency: console and operative times were longer with REVO-I (approximately 90 and 126 min, respectively) compared with da Vinci (50 and 92 min), reflecting the early learning curve of a novel platform [43]. Collectively, these findings indicate that REVO-I can be safely and effectively deployed for complex urologic oncology, achieving perioperative, oncologic, and functional results comparable to established robotic systems, albeit with some time penalties during its introduction phase.

The main limitations for REVO-I are its limited global availability and the lack of large-scale international validation studies. Without regulatory approvals in major markets outside Asia, its presence remains largely domestic. Future expansion will depend on international partnerships, rigorous comparative trials, and the development of a global training infrastructure.

### 3.5. Hinotori™ (Medicaroid)

Hinotori™, developed by the Japanese company Medicaroid (Kobe, Japan) in collaboration with Kawasaki Heavy Industries and Sysmex Corporation, is the first domestically produced multiport robotic system in Japan. The Hinotori surgical robot system consists of three components: the surgeon’s control cabin, the operating unit, and the vision unit. The system’s robotic arms are engineered for a wide range of motion with flexible port placement, reducing the risk of external collisions and allowing adaptation to various surgical approaches. The console’s immersive 3D HD vision supports precise dissection, while the instrument design provides multiple degrees of freedom for complex tissue manipulation. The patient-side cart accommodates four arms with 8 degrees of freedom and joint articulation designed to operate efficiently in confined pelvic spaces—an advantage for radical prostatectomy, which has been the system’s main clinical application to date.

Clinical evaluation of the Hinotori™ Surgical Robot has progressively expanded in Japan, with the majority of reports focusing on radical prostatectomy. Hinata et al. first demonstrated feasibility in a 30-patient RARP series, all completed without conversion and with a low adverse-event rate [44]. Nakayama et al. later confirmed the safety and reproducibility of 97 Hinotori RARPs, showing perioperative outcomes comparable to da Vinci cases and no Clavien–Dindo ≥III complications, while Yamada et al. reported the first Retzius-sparing series (6 Hinotori vs. 14 da Vinci), with equivalent continence recovery and oncologic outcomes, albeit with slightly longer console times during the adoption phase [45,46]. Miyamoto et al. provided broader real-world evidence by analyzing 91 Hinotori oncologic procedures—including RARP, RAPN, radical nephrectomy, nephroureterectomy, adrenalectomy, and two RARC cases—versus 277 da Vinci procedures, finding no conversions, no unrecoverable device failures, and perioperative and early oncologic outcomes broadly comparable across systems [47]. In specific comparisons, Kanehira et al. examined step-specific operative times in 42 Hinotori versus 88 da Vinci RARPs and showed that although total operative and console times were longer with Hinotori (235 vs. 200 min; 168.5 vs. 142.5 min), demanding steps such as pelvic lymph node dissection and vesicourethral anastomosis were unaffected, indicating skill transferability from da Vinci [48]. Similarly, Morizane et al. compared 52 Hinotori, 81 da Vinci, and 16 Hugo RARPs, finding no significant differences in complication rates, but shorter docking and console start times with da Vinci, underscoring efficiency gaps in workflow [49]. Beyond prostatectomy, Hinotori has been applied to more complex cases: Hayashi et al. reported the first RARC with intracorporeal diversion, while Motoyama et al. described the first radical nephroureterectomy, both completed safely with negative margins and no major complications [50,51]. Fukumoto and Kubota further extended its use to benign pelvic surgery with sacrocolpopexy, achieving outcomes comparable to da Vinci despite modestly longer operative times [52,53].

### 3.6. Senhance™ (Asensus Surgical)

The Senhance™ Surgical System, produced by Asensus Surgical (formerly TransEnterix, Durham, NC, USA), offers several unique features that set it apart from other multiport robotic platforms. Most notably, it provides true haptic feedback, enabling surgeons to perceive tactile resistance during tissue manipulation—an aspect absent from most current systems. This feedback has potential safety advantages, particularly in delicate dissections. Another distinctive element is the eye-tracking camera control, which allows the surgeon to guide the laparoscopic view simply by moving their eyes within the console display. This feature can improve efficiency by reducing reliance on manual camera repositioning. The system employs an open-console design, facilitating direct communication with the operating team, and its modular arms can be arranged to fit a variety of surgical setups. Senhance™ is compatible with standard reusable laparoscopic instruments and trocars, which may significantly reduce per-case costs in high-volume centers. This compatibility also enables hybrid procedures that integrate conventional laparoscopy and robotic assistance within the same case.

Clinical data on the Senhance™ Surgical System in urology have been reported mainly for radical prostatectomy, with additional contributions from smaller series in other procedures. The largest single-institution experience to date is from Hudolin et al., who analyzed 200 extraperitoneal Senhance-assisted radical prostatectomies [54]. Median estimated blood loss was 250 mL, with eight conversions (six to laparoscopy, two to open surgery), and early complications were recorded in 15 patients, the majority minor [54]. Functional outcomes were encouraging, with 92.7% of patients achieving continence at short-term follow-up [54]. In Lithuania, Venckus et al. presented outcomes of 127 consecutive radical prostatectomies performed with Senhance [55]. They reported a median operative time of 180 min, median blood loss of 250 mL, and an overall complication rate of 11.8%, with only three Clavien–Dindo ≥III events [55]. The overall positive surgical margin (PSM) rate was 33.9%, higher in pT3 than in pT2 disease [55]. Beyond prostatectomy, Kaštelan et al. documented Senhance applications in both prostate cancer and upper tract surgery, including nephrectomy and adrenalectomy, showing perioperative outcomes comparable to standard laparoscopy [56]. In the same institutional environment, Kuliš et al. specifically compared Senhance extraperitoneal radical prostatectomy with conventional laparoscopic surgery, reporting similar perioperative results while highlighting the benefits of robotic ergonomics, haptic feedback, and cost efficiency related to the use of reusable instruments [57]. Extending beyond Europe, Lin et al. compared 65 Senhance with 120 da Vinci radical prostatectomies, finding no significant differences in blood loss, complications, or early functional outcomes. Anastomosis time was longer with Senhance, but the per-case cost was markedly lower (US $4170 vs. $7675) [58]. Further multicenter evidence from the TRUST registry, analyzed by Staib et al., included 3239 Senhance procedures across nine European centers—498 in urology, predominantly radical prostatectomy—and confirmed a favorable safety profile, with low conversion rates, rare device malfunctions, an overall adverse event rate of 3.9%, and no mortality, reinforcing the system’s real-world feasibility [59].

### 3.7. KangDuo

The KangDuo robotic surgical platform (Suzhou KangDuo Robot Co., Ltd., Suzhou, China) is designed as a master–slave system comprising three main components: an open surgeon console, a robotic cart with three operating arms, and a vision cart. The surgeon console incorporates two monitors: the lower screen provides real-time intraoperative three-dimensional (3D) visualization, while the upper display can be used to access navigation tools or auxiliary imaging, including 3D reconstructions derived from preoperative CT scans. The open-console configuration allows the surgeon to maintain an upright posture, reducing the risk of neck strain and fatigue during prolonged procedures.

Clinical evidence on the KangDuo (KD) robotic system in urology is steadily expanding, primarily from Chinese centers. The most comprehensive experience to date was reported by Xiong et al., who analyzed 110 consecutive cases across multiple indications, including partial nephrectomy (*n* = 28), radical prostatectomy (*n* = 41), and urinary tract reconstructions (*n* = 41) [60]. All procedures were successfully completed without conversion, and no major complications were observed. Perioperative performance was favorable, with median operative times ranging from 112.5 min for partial nephrectomy to 157 min for pyeloplasty, and median blood loss remaining low across procedures (10–50 mL) [60]. Functional and oncologic outcomes were also encouraging, with no positive margins in partial nephrectomy, a 39% positive surgical margin rate in radical prostatectomy without biochemical recurrence at 11 months, and high success rates for reconstructive cases (96% for pyeloplasty, 92% for ureterovesical reimplantation) [60]. Additional studies have reinforced these observations. Li et al. focused specifically on KD-assisted partial nephrectomy, documenting its technical feasibility and perioperative safety profile, with blood loss, operative times, and complication rates comparable to laparoscopic and robotic benchmarks [61]. Building on this, Li et al. conducted a comparative analysis of partial nephrectomy performed with KD versus the da Vinci Si, reporting similar perioperative outcomes and showing no significant differences in oncologic control or quality-of-life recovery during medium-term follow-up [62]. Zhang et al. extended applications to upper tract oncologic surgery with robot-assisted nephroureterectomy, and in their comparative study with da Vinci, perioperative outcomes were broadly equivalent, underscoring KD’s suitability for complex oncologic resections [63]. More recent series have addressed technically demanding procedures. Feng et al. demonstrated the feasibility of KD in radical cystectomy with intracorporeal diversion comparing KD to Da Vinci Xi [64]. Among 34 patients (16 KD, 18 Da Vinci), all procedures were completed robotically, with a 100% surgical success rate and no significant differences across intraoperative, pathological, or postoperative outcomes [64]. Finally, the largest comparative analysis between Kangduo and Da Vinci, by Liu et al. included 201 urologic procedures and found that, although KD was associated with slightly longer operative times and drainage duration compared with da Vinci, it showed lower postoperative infection (11.7% vs. 29.1%) and fever rates (15.0% vs. 30.5%), with all other short-term outcomes equivalent [65].

### 3.8. MicroHand S

The Micro Hand S surgical robot, (Shandong WEGO Surgical Robot Co., Ltd., Weihai, China) introduced in 2013 through a collaboration between Central South University and Tianjin University, was conceived as a compact, lower-cost alternative for minimally invasive procedures. The platform operates in a master–slave configuration, comprising a surgeon’s console and a patient-side cart. At the console, surgeons interact with the system through a stereoscopic display, two master hand controllers, a multifunctional control panel, and four programmable foot pedals. Hand movements are translated into electrical signals that govern the instruments’ position, orientation, and grip within the operative field. The pedals are assigned to functions such as activating the system, controlling the endoscope, managing instrument disengagement, and applying electrosurgical energy, while the control panel is used for initialization and parameter setting before surgery. The patient-side cart incorporates a vertical column and a passive arm with an adjustable swivel head, which rotates up to ±90° and supports three active manipulators—two for instruments and one for the endoscope. Each manipulator is mounted on passive translational joints that permit precise alignment during setup, with additional orthogonally arranged passive joints that adapt automatically to the trocar fulcrum during use. This design enables the system to meet the geometric constraints of minimally invasive surgery while maintaining stable motion. By combining passive alignment mechanisms with active manipulators, the system facilitates rapid preoperative configuration and efficient intraoperative performance.

The initial clinical use of the Micro Hand S robot was reported in China between 2014 and 2015, involving a small cohort of patients undergoing procedures such as gastric perforation repair, appendectomy, cholecystectomy, and right colectomy [66]. To date, most published clinical experiences with the Micro Hand S system have been concentrated in general surgery, particularly in colorectal and gastric procedures [67,68,69]. By contrast, experience with the Micro Hand S system in urology remains very limited. The only published study to date reported its use in robot-assisted laparoscopic radical nephrectomy performed via 5G-enabled telesurgery. In this series, 29 patients across eight primary hospitals underwent remote nephrectomy controlled from a tertiary center, with a 100% success rate and no major intra- or postoperative complications [70]. The findings demonstrated both the technical feasibility and safety of combining the Micro Hand S platform with advanced telecommunication technologies to extend specialist surgical care to peripheral hospitals.

### 3.9. Dexter™ (Distalmotion)

The Dexter™ Surgical System (Distalmotion, Lausanne, Switzerland) is an open, modular robotic platform designed to integrate seamlessly with existing laparoscopic infrastructure. It comprises an open ergonomic console, two mobile instrument carts each carrying a robotic arm, and a dedicated endoscope cart. The console is equipped with clutch and endoscope pedals, enabling intuitive control of the instruments and adjustment of the visual field. Each arm provides seven degrees of freedom with an angulation of up to 75°, supporting a range of reusable instruments such as monopolar scissors, monopolar hooks, bipolar Maryland dissectors, bipolar graspers, and needle holders. A defining feature of Dexter is its compact and mobile architecture: all modules can be easily transferred between operating rooms, stored efficiently, and positioned to preserve ample space around the operating table for the assistant. The system is an open platform, compatible with standard laparoscopic trocars and existing hospital equipment, including 3D and fluorescence imaging, energy devices, and endoscopic systems—such as the Karl Storz TipCam^®^1 S 3D (Karl Storz SE & Co. KG, Tuttlingen, Germany) used in early clinical applications. Importantly, Dexter enables hybrid procedures, allowing rapid transitions between conventional laparoscopy and robotic-assisted surgery without undocking. The robotic arms can be folded into a compact “laparoscopic mode” within seconds, maintaining patient access while keeping the surgeon sterile thanks to disposable console covers. This flexibility permits intraoperative escalation, whereby initial mobilization may be completed laparoscopically and the procedure finalized robotically. Instrument handles are reusable and designed for standard reprocessing, further supporting cost efficiency.

The first urological procedures performed with the Dexter system—a radical and a simple prostatectomy—were carried out in Bern in June 2022 by Dr. Dominik Böhlen, marking the clinical introduction of this platform in urology [71]. Shortly thereafter, the first structured evaluation came from a French group, who reported a feasibility series of ten patients undergoing robot-assisted radical prostatectomy with or without extended lymphadenectomy [72]. All procedures were completed without conversion or device-related complications, establishing the system’s technical reliability and confirming the practicality of its open-console design and hybrid laparoscopic–robotic workflow. Building upon this early experience, the same team subsequently evaluated a larger cohort of 47 consecutive radical prostatectomies performed between April 2022 and May 2023 [73]. In this series, intraoperative performance was consistent, with no complications, conversions, or major technical failures. Operative efficiency proved acceptable, with a median duration of 198 min, while the short median hospital stay of 2 days reflected a rapid postoperative recovery [73]. Oncological safety appeared encouraging, with positive surgical margins identified in 17% of patients and only two cases showing biochemical recurrence during short-term follow-up [73]. Functional outcomes were also favorable, as 94% of patients achieved urinary continence within three months, and just over half regained sexual function by the same interval [73]. Taken together, these studies provide the first structured evidence supporting the feasibility, safety, and short-term effectiveness of the Dexter system in radical prostatectomy, situating it among the emerging alternative robotic platforms for urologic surgery. Beyond urology, early experiences from visceral surgery departments, such as those reported by Conrad et al., have demonstrated that the Dexter system can be safely implemented for procedures of low to medium complexity, further underscoring its versatility [74].

### 3.10. Toumai^®^ (MedBot)

The Toumai MT-1000 robotic system (MicroPort MedBot, Shanghai, China) integrates several advanced components aimed at enhancing surgical precision and safety. It is equipped with four robotic arms and a stereoscopic platform that provides surgeons with an immersive naked-eye 3D view, supplemented by dual-console capability, simulation functions, and convenient features such as picture-in-picture display and one-click arm expansion or retraction. A key innovation of the system is its refined force perception technology, which enables accurate tactile feedback during procedures. With a high-frequency response of 4000 Hz and rapid reaction time of 250 μs, it allows surgeons to perform delicate maneuvers with greater control. Real-time visualization is supported by FPGA-based image processing and dual fiber optic transmission, reducing latency to under 50 ms and ensuring timely, high-resolution imaging (1920 × 1080 at 60 Hz). Additional visual optimization tools, including an intelligent smoke removal algorithm and vascular enhancement mode, further improve clarity of the operative field. The robotic instruments, measuring ≤8.4 mm in diameter, provide seven degrees of freedom, 540° rotation, and stable energy delivery for precise dissection and suturing. From a safety perspective, the Toumai MT-1000 incorporates automatic protective mechanisms such as console lockout when the surgeon’s head leaves the viewer, as well as foot pedal safeguards to prevent accidental activation. Notably, the platform has pioneered technologies such as 5G-enabled remote operation with a master–slave delay of ≤70 ms, positioning it among the most technologically advanced surgical systems currently available.

The first prospective clinical experience with the Toumai^®^ robotic system in urology was reported by Tan et al. who described 36 procedures including radical and partial nephrectomy, nephroureterectomy, adrenalectomy, and radical prostatectomy [75]. All surgeries were successfully completed without conversion, major intraoperative complications, or technical failures, demonstrating the feasibility of the platform across different urologic indications. Shortly thereafter, Pokhrel et al. presented a smaller but detailed prospective series of 20 patients—17 undergoing various nephrectomy procedures and three radical prostatectomies—performed at a single center in Zhengzhou [76]. Their report confirmed the safety and feasibility of the system, with no conversions and only one major complication, while perioperative outcomes, renal function preservation, and early continence recovery were satisfactory. Building on these early results, Pokhrel et al. published the first focused prospective evaluation of the Toumai^®^ system in partial nephrectomy [77]. In this single-center study, 11 patients underwent robot-assisted partial nephrectomy, all completed successfully without conversion or major technical malfunctions. Median operative time was 107 min, docking time 8 min, and estimated blood loss 50 mL. A warm ischemia time of 9 min was achieved in clamped cases, while one off-clamp procedure was also successfully performed [77]. All patients had negative surgical margins, stable postoperative renal function, and no 30-day readmissions. The authors highlighted the system’s advanced features—including haptic force feedback, high-frequency response, and enhanced imaging technologies—as key contributors to surgical precision and efficiency.

In parallel, preclinical investigations have also expanded knowledge of Toumai’s potential applications. Sighinolfi et al. described the first Italian preclinical experience, where the system was tested in a porcine model for both telesurgery and complex reconstructive procedures [78]. A telesurgical left radical nephrectomy was successfully performed over a distance of nearly 100 km with <200 ms latency and minimal frame loss, demonstrating the platform’s 5G-enabled remote surgery capability [78]. Additional procedures, including radical cystectomy with extended lymphadenectomy and ileal neobladder reconstruction, were completed without intraoperative complications, with operative times of 70 and 60 min, respectively. Most recently, Beatrici et al. reported the first European clinical telesurgery using the Toumai^®^ system [79]. A robot-assisted radical prostatectomy was performed remotely between two Belgian centers located 25 km apart. The operation was completed without conversion, technical failure, or intraoperative complications. Network performance was optimal, with a latency of 20 ms, jitter <5 ms, and no signal loss, while surgeon feedback indicated high system usability and low workload [79]. The patient was discharged on postoperative day four and had regained satisfactory urinary continence at first follow-up. This landmark case provides the first human evidence in Europe that complex urologic surgery can be safely performed remotely with the Toumai^®^ platform, underscoring its potential role in the future integration of telesurgery into routine practice.

## 4. Economic Considerations and Cost Models

Economic feasibility remains a major determinant of robotic platform adoption, particularly for hospitals in resource-constrained settings. The da Vinci system, while clinically validated, carries one of the highest capital expenditures in robotic surgery, with acquisition costs often exceeding USD 2 million and substantial ongoing maintenance fees, compounded by the proprietary nature of its instruments [8].

Several emerging platforms have introduced strategies to reduce overall costs. The Senhance system employs standard reusable laparoscopic instruments, resulting in a substantially lower per-case cost, with reports showing a mean cost of USD 4170 per Senhance radical prostatectomy compared with USD 7675 for da Vinci cases [58]. Similarly, the Dexter system emphasizes cost efficiency through reusable instruments and compatibility with existing laparoscopic infrastructure, reducing the need for additional equipment [73].

In contrast, the Avatera platform uses exclusively single-use instruments, eliminating reprocessing requirements and potential damage costs but shifting economic considerations toward consumables [35,39]. The REVO-I system offers lower acquisition and maintenance costs than international competitors, which may be advantageous in domestic or regional markets [43].

Although explicit comparative cost data remain limited for other systems, modular and mobile architectures (e.g., Versius and Hugo) are designed to optimize operating room utilization and efficiency, which may indirectly influence cost-effectiveness. Publicly available acquisition prices are scarce and vary significantly across regions and institutions, so published cost-effectiveness analyses remain the most reliable source for comparison at this stage. Further independent evaluations will be essential to guide procurement decisions as these platforms mature and gain regulatory approval.

## 5. Future Perspectives

The current evidence base for next-generation robotic platforms in urology remains highly heterogeneous, reflecting differences in regulatory approval, geographic distribution, and stage of commercialization. Systems such as Hugo™ RAS and Versius™ have been evaluated through multicenter series and early comparative studies, providing a relatively robust body of literature. In contrast, other platforms—including Avatera™, REVO-I, and MicroHand S—are supported primarily by single-institution reports or preclinical investigations, underscoring their early developmental stage. This variability highlights the importance of ongoing research efforts, particularly multicenter trials, long-term oncologic and functional outcome studies, and independent cost-effectiveness analyses, to inform evidence-based adoption. Emerging evidence also underscores the importance of structured, long-term training programs to achieve proficiency in robotic procedures such as radical prostatectomy, with data showing that at least 12 months of exposure significantly improves outcomes and supports the use of standardized tools to evaluate learning curves [80]. Against this backdrop, several emerging trends point toward a transformative future for robotic surgery in urology.

The rapid evolution of robotic platforms in urologic surgery heralds a transformative era defined by technological diversification, greater cost accessibility, and new models of surgical care. Next-generation systems are no longer limited to replicating da Vinci’s capabilities but increasingly introduce distinctive innovations that may reshape clinical practice. Artificial intelligence and machine learning are expected to become integral to robotic consoles, providing real-time intraoperative guidance, predictive analytics for complications, and automated performance metrics to standardize surgical quality [81,82]. Similarly, the advent of telesurgery—exemplified by platforms like Toumai^®^ with 5G-enabled connectivity—has the potential to dissolve geographical barriers, extending specialist expertise to underserved regions through remote mentoring and proctoring. Another key trajectory is miniaturization, with the development of single-port and reduced-port systems that aim to enhance cosmesis and reduce invasiveness while addressing challenges of triangulation and instrument crowding. At the same time, cost-containment strategies such as reusable instruments (Dexter™), modular architectures (Versius™), and competitive pricing models are poised to broaden global access, particularly in resource-limited settings. Parallel to these advances, outpatient and ambulatory robotic surgery—leveraging systems with rapid docking and turnover times—may redefine perioperative care pathways in select urologic procedures.

Despite this momentum, challenges remain. Training frameworks must adapt to accommodate platform heterogeneity and ensure surgeon proficiency without compromising patient safety. Regulatory agencies will be tasked with balancing streamlined approvals for new technologies, particularly AI-driven functionalities, against the need for rigorous efficacy and safety standards. Finally, robust comparative trials and long-term oncologic and functional outcome data remain essential to justify widespread adoption and institutional investment. As robotic surgery transitions from monopoly to plurality, collaboration among surgeons, engineers, and policymakers will be critical to harness innovation while maintaining equitable, value-based care.

## 6. Conclusions

Robotic urologic surgery has entered a transformative phase, with next-generation systems now showing outcomes comparable to earlier dominant platforms. These technologies—offering advantages in portability, cost, and feedback—signal a shift toward tailoring robotics to surgical needs rather than adapting workflows to a single model. Early results are promising, but widespread adoption will depend on multi-institutional validation, scalable training, and transparent cost-effectiveness analyses. The success of this era of robotic “pluralism” will depend less on novelty than on expanding access without compromising quality. As artificial intelligence, telesurgery, and miniaturization evolve, urologists must guide their integration into practice. The ultimate goal is to ensure that robotic innovation bridges precision with affordability and extends expertise to underserved populations while upholding the principles of safe, effective patient care.

## Figures and Tables

**Table 1:** Key Features of Next-Generation Robotic Platforms in Urology.

Platform	Manufacturer	Regulatory Approval (CE Mark/FDA)	Country of Origin	Key Features and Design Philosophy
Hugo™ RAS	Medtronic	CE 2021; FDA -	USA	Modular, wheeled independent arms; open console; emphasizes flexibility and/or adaptability.
Versius™	CMR Surgical	CE 2019; FDA 2024	UK	Compact, modular arms on wheeled units; open console; ergonomic design for laparoscopic transition.
Avatera™	Avateramedical GmbH	CE 2019; FDA -	Germany	Exclusively single-use instruments; closed console; aims to simplify workflow and sterilization.
REVO-I	Meere Company Inc.	No CE; FDA -	South Korea	Design mimics da Vinci to reduce learning curve; closed console; reusable instruments.
Hinotori™	Medicaroid	No CE; FDA -	Japan	First Japanese multiport system; engineered for wide range of motion in confined spaces.
Senhance™	Asensus Surgical	CE 2016; FDA 2017	USA	True haptic feedback; eye-tracking camera control; uses standard laparoscopic instruments.
KangDuo	Various	No CE; FDA -	China	Open console; dual-screen display (3D surgery + auxiliary imaging); master–slave system.
MicroHand S	Central South/Tianjin Uni	No CE; FDA -	China	Compact, low-cost; designed for rapid setup; pioneered 5G telesurgery in urology.
Dexter™	Distalmotion	CE 2020; FDA -	Switzerland	Hybrid laparoscopic–robotic platform; arms fold for rapid switch to laparoscopy; open platform.
Toumai^®^	MedBot	CE 2024; FDA -	China	Naked-eye 3D view; advanced force feedback; 5G telesurgery capabilities; dual-console.

## Data Availability

Data sharing is not applicable.
